# MoS_2_ Channel‐Enhanced High‐Density Charge Trap Flash Memory and Machine Learning‐Assisted Sensing Methodologies for Memory‐Centric Computing Systems

**DOI:** 10.1002/advs.202501926

**Published:** 2025-06-10

**Authors:** Ki Han Kim, Ju Han Park, Khang June Lee, Ji‐Won Seo, Yeong Kwon Kim, Junhwan Choi, Min‐Jae Seo, Byung Chul Jang

**Affiliations:** ^1^ School of Electronic and Electrical Engineering Kyungpook National University 80 Daehakro, Bukgu Daegu 41566 Republic of Korea; ^2^ School of Semiconductor Convergence Engineering Kyungpook National University 80 Daehakro, Bukgu Daegu 41566 Republic of Korea; ^3^ Department of Electronic Materials Engineering The University of Suwon Hwaseong Gyeonggi‐do 18323 Republic of Korea; ^4^ School of Advanced Fusion Studies University of Seoul Seoulsiripdae‐ro 163, Dongdaemun‐gu Seoul 02504 Republic of Korea; ^5^ Department of Chemical Engineering Dankook University 152 Jukjeon‐ro, Suji‐gu Yongin Gyeonggi‐do 16890 Republic of Korea

**Keywords:** 3D NAND Flash, machine learning, MoS_2_, nonvolatile memory

## Abstract

Driven by the shift of artificial intelligence (AI) workloads to edge devices, there is a growing demand for nonvolatile memory solutions that offer high‐density, low‐power consumption, and reliability. However, well‐established 3D NAND Flash using polycrystalline Si (Poly‐Si) channel encounters bottlenecks in increasing bit density due to short‐channel effects and cell‐current limitations. This study investigates molybdenum disulfide (MoS_2_) as an alternative channel material for 3D NAND Flash cells. MoS_2_’s low bandgap facilitates hole‐injection‐based erase, achieving a broader memory window at moderate voltages. Furthermore, adopting a low‐*k* (≈2.2) tunneling layer improves the gate‐coupling ratio, reducing program/erase voltages and enhancing reliability, with endurance up to 10^4^ cycles and retention of 10^5^ s. Comprehensive analyses, including thickness‐dependent MoS_2_ electrical measurements, temperature‐dependent conduction studies, and Technology Computer‐Aided Design (TCAD) simulations, elucidate the relationship between channel thickness and reliability metrics such as endurance and retention. Furthermore, deep reinforcement learning–driven Berkeley Short‐channel IGFET Model (BSIM) parameter calibration enables seamless integration of the MoS_2_ model with a fabricated page‐buffer chip, allowing circuit‐level verification of sensing margins. This methodology can be applicable to new channel materials for next‐generation memory devices. These results demonstrate that MoS_2_‐based nonvolatile memory effectively meets high‐density, low‐power, and reliable storage needs, presenting a promising solution for AI‐centric edge computing.

## Graphical abstract

Driven by AI computing demands, this study investigates MoS_2_ channels for 3D NAND Flash to achieve high‐density, low‐power, and reliable nonvolatile memory. MoS_2_ enables a large memory window and lower operating voltages with low‐*k* tunneling layer, demonstrating endurance of 10⁴ cycles and retention of 10⁵ s. Comprehensive analyses and machine learning‐assisted sensing validation confirm potential of MoS_2_‐based memory for AI‐centric memory solutions.

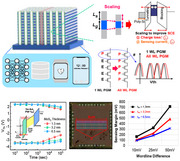

## 1 Introduction

The rapid proliferation of AI technology across various industries has revolutionized computing paradigms, driving a shift toward decentralization in AI processing. Conventional AI systems, which rely heavily on cloud‐based data centers for computation, face key issues such as privacy concerns, latency, and connectivity limitations.^[^ ^1^ ^]^ To address these issues, on‐device AI edge technology has emerged as a transformative solution, enabling real‐time AI task execution directly on smartphones and Internet of Things (IoT) edge devices.^[^ ^2^ ^]^ The on‐device AI can alleviate privacy concerns associated with data transfer to the cloud by integrating AI workloads into edge devices where data is initially generated. Consequently, the widespread adoption of on‐device AI underscores the need for high‐density, low‐power nonvolatile memory devices to efficiently process and store the massive volumes of locally generated data.

**Figure 1:**
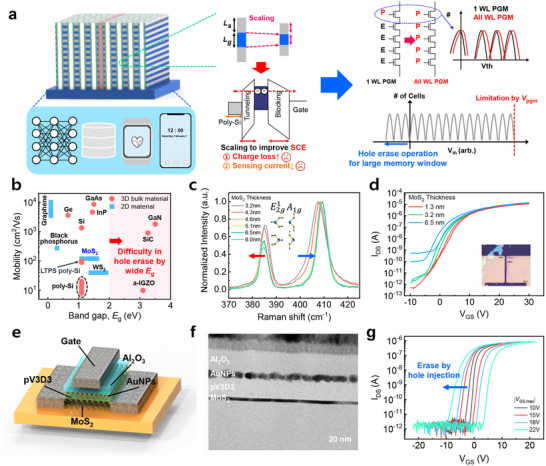
a) Schematic of 3D NAND Flash which can improve the system performance of edge devices. To increase bit density of 3D NAND Flash memory, the high carrier mobility and low bandgap channel material is required to achieve a large memory window and alleviate cell‐induced issues. b) Summary of the mobility versus bandgap for a variety of bulk and nanomaterials‐based semiconductors at room temperature. The data were redrawn from the references.^[^ ^23^ ^]^ c) Raman spectra for MoS_2_ flake with various thickness. d) Electrical characteristics of the MoS_2_‐based device with channel thicknesses of 1.3, 3.2, and 6.5 nm. e) Schematic illustration of the fabricated MoS_2_‐based synaptic memory device. f) A cross‐sectional high‐resolution TEM image of the gate stack of the fabricated memory device. g) Electrical characteristics of the MoS_2_‐based memory device under varying *V*_GS_ sweep range.

3D NAND Flash memory, a widely used storage technology in data center, wearable devices, and smartphones, is well suitable for meeting these demands. To achieve higher bit densities, the number of word‐line (WL) layers in 3D NAND Flash must be increased. Over the last decade, commercial 3D NAND products have already featured up to 300 word‐lines (WLs),^[^ ^3^ ^]^ and it is predicted that more than 1000 WLs will be realized in the near future.^[^ ^4^ ^]^ However, the increasing WLs in 3D NAND cause significant challenges, including the degraded sensing current due to increased string resistance and the burden on etch process for 3D structure formation.^[^ ^5^ ^]^ To alleviate the burden on etch process, the gate length (*L*_g_) and spacer length (*L*_s_) have been scaled down, exacerbating of short‐channel effects (SCEs).^[^ ^5^, ^6^ ^]^ To improve the SCEs, it is inevitable to thin channel layer rather than tunneling oxide‐Si_3_N_4_ trap‐blocking oxide (ONO) layers to enhance gate controllability by reducing the equivalent oxide thickness, as scaling the ONO layers results in vertical charge loss (**Figure** **1**a). However, alongside the trend of increasing string resistance in 3D NAND, the scaling of the channel layer accelerates the reduction in cell current, resulting in sensing challenges. Furthermore, insufficient cell current leads to back‐pattern dependency (BPD)‐induced threshold voltage (*V*_Th_) variability issue, thereby narrowing the memory read window.^[^ ^7^ ^]^ Because cells within the same string are influenced by previously programmed states, *V*_Th_ increases in cells programmed earlier as WL‐by‐WL programming progresses. This arises from the IR drop caused by increasing array loading resistance, which changes the virtual source or drain potential and shifts *V*_Th_ accordingly.^[^ ^7^ ^]^ These issues are aggravated in current 3D NAND cell device due to the polycrystalline silicon (Poly‐Si) channel material, which includes defective grain boundaries (GBs) with numerous trap sites.^[^ ^8^ ^]^ As the *L*_g_ shrinks, Poly‐Si channel suffers more pronounced SCEs and GB‐related interface traps, making them increasingly unsuitable for development of higher density 3D NAND Flash memory. Therefore, introducing channel materials with higher carrier mobility than conventional Poly‐Si is essential for advancing 3D NAND Flash to meet the rising data volume driven by AI innovations.

Among various alternatives, transition metal dichalcogenides (TMDs)—particularly molybdenum disulfide (MoS_2_)—have garnered significant attention as promising channel materials for scaled transistor.^[^ ^9^ ^]^ In contrast to conventional Si, MoS_2_ features an atomically thin structure with high carrier mobility, strong immunity to SCEs, and atomically sharp surfaces without dangling bonds. Unlike graphene, which has no bandgap, monolayer MoS_2_ exhibits a bandgap of 1.8 eV, making it suitable for electronic devices. MoS_2_‐based transistors demonstrated high on/off current ratios, making them well‐suited for memory applications that require large memory windows and multi‐bit precision (>5 bits).^[^ ^10^ ^]^ Moreover, because memory window in 3D NAND Flash is constrained by the program voltage (*V*_PGM_) generated by the voltage generator circuitry, the narrow bandgap of MoS_2_ compared to an equivalent ultrathin Si film facilitates the hole injected‐erase operation, thereby expanding the memory window in the negative *V*_T_ direction (Figure 1b). Recent TCAD simulations have demonstrated that the inherent properties of MoS_2_ improve both memory windows and memory operational efficiency in scaled *L*_g_ and *L*_s_ compared to poly‐Si channels in 3D NAND Flash.^[^ ^11^ ^]^ Although inherent MoS_2_ material is suitable as channel layer for developing 3D NAND Flash memory, it is necessary to investigate in‐depthly memory characteristics, reliability, and sensing capability such as sensing margin according to channel layer thickness for practical application. Understanding these factors will be crucial for bringing the benefits of MoS_2_ channels to the next generation of high‐density, low‐power 3D NAND Flash memory technologies that support on‐device AI and beyond.

In this study, we systematically investigate the electrical characteristics of MoS_2_ with various thicknesses as a high‐mobility alternative to conventional poly‐Si channel in 3D NAND Flash. By employing a low‐*k* (≈2.2) poly(1,3,5‐trimethyl‐1,3,5‐trivinyl cyclotrisiloxane) (pV3D3) tunneling dielectric—deposited via solvent‐initiated chemical vapor deposition (iCVD)^[^ ^12^ ^]^—we enhance the gate‐coupling ratio and thus reduce the operating voltages required for program and erase operations. Through comprehensive electrical characterization for MoS_2_ channel thickness, we analyze how MoS_2_ channel thickness impacts memory performance (program/erase speeds), reliability (endurance, retention), and the underlying device physics, supported by experimental measurements, TCAD simulations, and temperature‐dependent conduction analysis. Finally, by integrating the device‐level characteristics into a circuit‐level framework—using deep reinforcement learning for BSIM parameter optimization and a fabricated page‐buffer chip—we validate the sensing margins and circuit‐level behavior of 3D NAND Flash memory. This extensive investigation underscores the feasibility of MoS_2_ as an innovative channel material, paving the way for memory‐centric AI applications at the edge device.

## 2 Results and Discussion

Figure 1a shows schematically the 3D NAND Flash memory in edge devices for on‐device AI applications. By processing data locally near the data generation source, edge computing effectively reduces latency, optimizes bandwidth utilization, and enhances privacy. The advanced sensors (e.g., healthcare and image sensors), AI accelerator, and storage elements like 3D NAND Flash are integrated into the edge devices such as smartphone and wearable devices. In these edge devices, sensor data must be rapidly accessed and processed by AI accelerators, making high‐density, energy‐efficient, and reliable 3D NAND Flash indispensable. The sensors gather diverse physical and biological data in real‐time, serving as inputs for AI algorithms, while the AI accelerator implements real‐time data processing and rapid decision‐making. To boost system efficiency, optimization of 3D NAND Flash storage is imperative, because all synapse weight and sensor output data are stored and processed in an energy‐efficient manner within the pre‐trained system with high‐density storage. As a well‐established nonvolatile memory technology, 3D NAND Flash memory achieves exceptionally high‐density, enabling large‐scale storage of synapse weights needed for AI accelerator's operations and thereby improving data throughput. However, to further increase WL stacking and realize high‐density, reliable 3D NAND cell device, the following reliability issues must be resolved: i) insufficient cell current‐induced BPD and sensing issue, and ii) the need of a large memory window for multi‐bit per cell technology. Among semiconductor materials, the MoS_2_ material is a promising candidate to address these challenges due to its low bandgap, high mobility, and ultraclean surface with dangling bond‐free at an ultrathin body, as shown in Figure 1b. These features enable hole injection‐based erase for a larger memory window, high on‐currents, and improved transconductance.

To investigate MoS_2_ channel thicknesses, we utilized Raman spectroscopy, a well‐known non‐destructive technique for film characterization. Figure 1c shows the Raman spectra for different MoS_2_ thicknesses. As the thickness of MoS_2_ increases, the *A*_1g_ mode associated with the out‐of‐plane vibration of S atoms exhibits a blue shift, while the *E* ^1^_2g_ mode related to the in‐plane vibrations of Mo and S atoms shows a red shift.^[^ ^13^ ^]^ These shifts arise from the stronger van der Waals interactions between layers, which stiffen the *A*_1g_ mode by the increased out‐of‐plane interlayer coupling and soften the *E* ^1^_2g_ mode. This makes Raman spectroscopy a valuable, non‐destructive method for monitoring layer thickness in large‐area MoS_2_ films, eliminating the need for destructive transmission electron microscopy (TEM) analysis—an advantage for industrial‐scale mass production.

Figure 1d shows the electrical characteristics of MoS_2_ transistors with different channel thickness. Compared to the 1.3 nm‐thick MoS_2_ transistor, the 6.5 nm‐thick MoS_2_ exhibits degraded subthreshold swing (*SS*), a lower threshold voltage (*V*_Th_), and a higher off‐current. This results from the partially depleted body of the thicker MoS_2_ channel, as will be discussed in more detail. To scrutinize memory performance with respect to MoS_2_ channel thickness, we fabricated a high gate‐coupling ratio memory device using the MoS_2_ channel and Au nanoparticles (AuNPs) as the charge storage layer (Figure 1e). In this work, we employed a mechanically exfoliated MoS_2_ material as channel layer to investigate its inherent properties. To lower the operating voltage by increasing gate‐coupling ratio (*C*_Block_/(*C*_Block_+*C*_Tunnel_)),^[^ ^14^ ^]^ we used a low‐*k* (≈2.2) poly(1,3,5‐trimethyl‐1,3,5‐trivinyl cyclotrisiloxane) (pV3D3) tunneling dielectric deposited via solvent‐initiated chemical vapor deposition (iCVD). Even at sub‐10 nm thicknesses, the pV3D3 dielectric exhibited exceptional dielectric properties, such as ultralow leakage (below 10^−9^ A cm^−2^ at 3 MV cm^−1^), a high bandgap (≈8.25 eV), low dielectric constant (≈2.2), excellent chemical stability, and strong resistance to mechanical stress.^[^ ^12^ ^]^ AuNPs were adopted as the charge storage medium thanks to their high work function (≈5.2 eV) and discrete spatial distribution. These features create a deep potential well that minimizes both vertical and lateral charge loss, thereby improving retention. AuNPs with a discrete spatial distribution can be easily deposited using a thermal evaporator, since they spontaneously form the nanoparticle morphologies to reduce surface energy in the absence of sufficient Au for continuous film formation. As shown in Figure 1f, the cross‐sectional high‐resolution TEM (HRTEM) image of the fabricated memory device confirms the well‐formed ultrathin pV3D3 tunneling layer (≈13 nm), Al_2_O_3_ blocking layer (≈20 nm), and AuNPs on the dangling bond‐free MoS_2_ channel surface. Furthermore, the enlarged HRTEM image indicates that the ultraclean interface between the pV3D3 tunneling layer and MoS_2_ channel surface (Figure S1, Supporting Information). This indicates that iCVD‐deposited pV3D3 induces minimal lattice distortion or oxidation at the MoS_2_ surface, further supported by X‐ray photoelectron spectroscopy (XPS) analyses (Figure S2, Supporting Information). Figure 1g presents representative electrical characteristics of our memory device with a 3.2 nm‐thick MoS_2_ channel. The ultraclean interface and discrete charge storage of AuNPs allow MoS_2_ memory device to achieve memory characteristics with a high on/off ratio. Under sufficiently large positive *V*_PGM_​​, programming occurs as electrons are injected into AuNPs via Fowler–Nordheim (F–N) tunneling, causing a positive shift in *V*_Th_. Conversely, applying a negative erase voltage (*V*_ERS_) shifts *V*_Th_ ​ into more negative values, demonstrating hole injection‐based erase operation. Owing to the relatively small valence band offset between MoS_2_ with the electron affinity of 4.2 eV^[^ ^15^ ^]^ and pV3D3 with the lowest unoccupied molecular orbital level of 1.2 eV,^[^ ^12^ ^]^ hole injection into the AuNPs is facilitated, further extending the memory window (See Figure S3, Supporting Information, for energy band diagram). Altogether, our MoS_2_ memory device shows considerable potential for developing high‐density nonvolatile memory with multi‐bit per cell technology.

**Figure 2:**
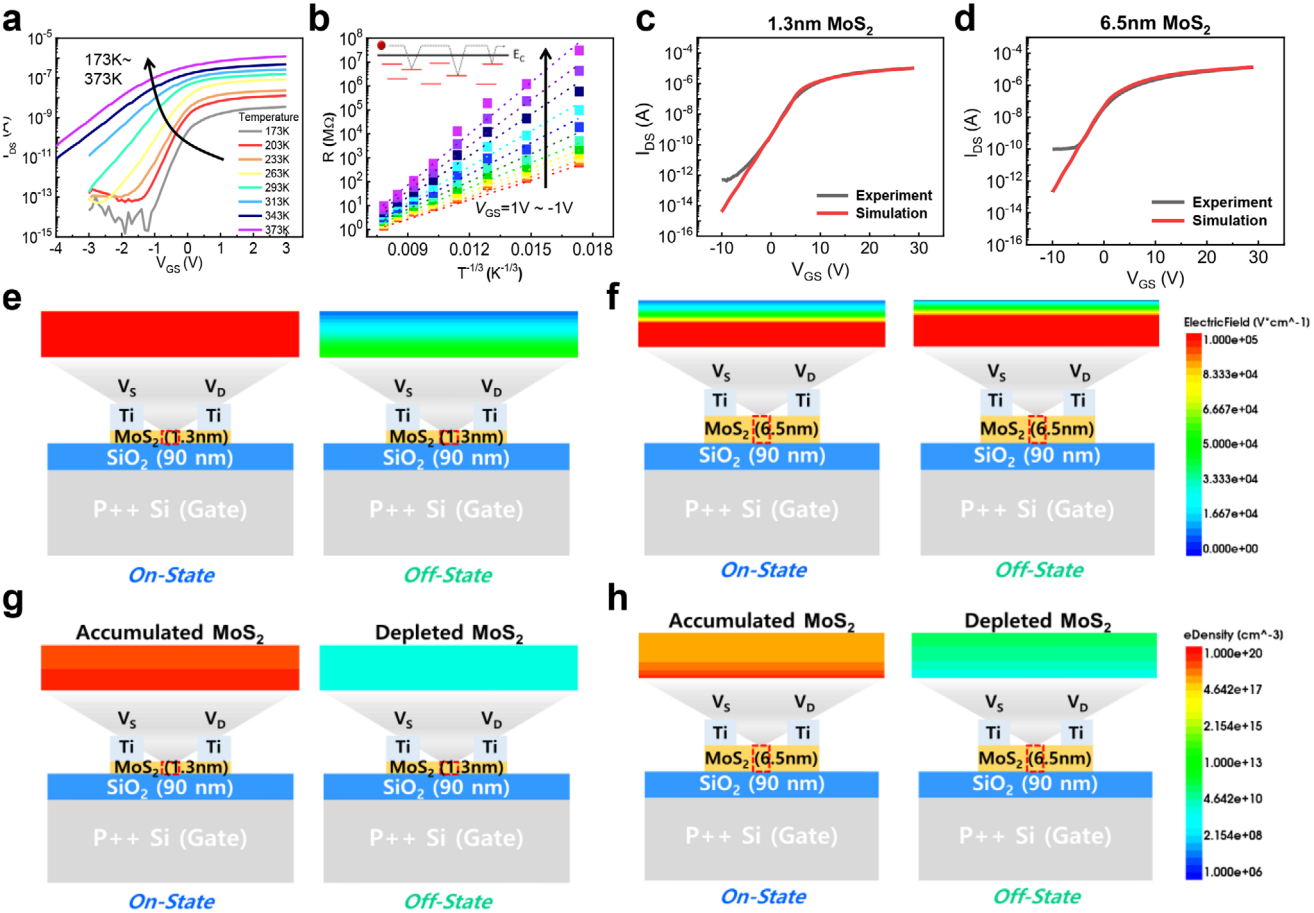
a) MoS_2_‐based device as a function of temperature. b) Temperature‐dependent variations in channel resistance at low *V*_DS_ of MoS_2_‐based device to investigate the conduction mechanism in MoS_2_. The calibrated TCAD simulation results for MoS_2_ devices with thickness of c) 1.3 nm and d) 6.5 nm. The simulated electric field distributions in MoS_2_ channel layer during the on and off states for e) 1.3 nm‐thick and f) 6.5 nm‐thick devices. The simulated electron density in MoS_2_ channel layer during the on and off states for g) 1.3 nm‐thick and h) 6.5 nm‐thick devices.

To analyze the conduction mechanism in MoS_2_ channel, we investigate the temperature dependence of electrical characteristics of MoS_2_ memory device, as shown in **Figure** **2** **a**. Notably, as temperature rises, the on‐current increases, while the threshold voltage *V*_Th_​ decreases and the subthreshold swing (SS) worsens. This indicates that the dominant conduction in the strong accumulation region is governed not by phonon scattering but by another mechanism. Prior work has reported that electron transport in MoS_2_ follows a variable‐range hopping (VRH) mechanism.^[^ ^16^ ^]^ At low drain voltages (*V*_DS_​), the channel resistance in the linear regime can be described by $R\propto R_{0}\left[{\left(\frac{T_{0}}{T}\right)}^{1/3}\right]$, where *R*_0_ and *T*_0_ fit parameters. Figure 2b confirms that our device's channel resistance at *V*_DS_​ = 1 V fits well to the VRH model. In MoS_2_, VRH conduction occurs via sulfur vacancies (SVs) that serve as localized transport sites. At elevated temperatures, carriers gain sufficient energy to hop between SVs, increasing conductivity. Furthermore, we observe that *T*_0_ decreases with increasing gate voltage, indicating that the VRH contribution to charge conduction is mitigated under high gate voltage region (Figure S4, Supporting Information). Note that the sensing in NAND Flash is generally performed by evaluating *V*_Th_ via commercial sensing circuit such as page buffer, as will be discussed in more detail. Thus, The VRH conduction plays an important role in determining the sensing capability of MoS_2_ memory device. Although the dispersed SVs enhance charge transport, excessive SV density can lead to defect clustering, deep trapping levels that degrade electrical properties. Optimal SV density control via MoS_2_ synthesis technique such as chemical vapor deposition is therefore essential to balance defect‐induced reliability concerns with the on‐current necessary for robust sensing. In addition to VRH conduction, the temperature‐dependent conductivity is influenced by the charged‐impurity scattering, which are presumably associated with the substrate, generating the disordered potential landscape required for hopping conduction. Considering that 3D NAND Flash cell device is fabricated through channel last process, the charge‐impurity scattering could be mitigated by deposition of hexagonal boron nitride with low trap density^[^ ^17^ ^]^ on the backside of the channel after MoS_2_ channel formation.

In addition to analysis of conduction mechanism, we scrutinize the underlying device physics governing MoS_2_ channel thickness using the Technology Computer‐Aided Design (TCAD) simulation. We successfully calibrated the experimental I‐V curves for 1.3 and 6.5 nm MoS_2_ channels (Figure 1d), as shown in Figure 2c,d. Details of the material parameters used for this calibration are provided in Table S1 (Supporting Information). The discrepancy in off‐current between the experimental and simulated data arises from the resolution limits of our parameter analyzer. Figure 2e,f shows the gate electric‐field (E‐field) distributions under *V*_GS_ = 30 V (on‐state) and −10 V (off‐state) for 1.3 and 6.5 nm channel thicknesses, respectively. It is confirmed that the gate E‐field is confined near the top surface in the thicker channel, leading to partial depletion in the off‐state. Figure 2g,h present electron‐density (e‐density) under on‐ and off‐states for 1.3 and 6.5 nm channel thicknesses, respectively. Thinner channel shows better gate control over the channel, resulting in higher e‐density in on‐state and lower e‐density in off‐state, compared to thicker channels. Therefore, as MoS_2_ thickness increases, we demonstrate that *SS* degradation, reduced *V*_Th_, and increased off‐current in thicker MoS_2_ channel device arise from the partially depleted body and weaker gate controllability. Therefore, it is essential to investigate the effects of the thickness‐dependent electrical characteristics of the MoS_2_ channel on memory performance and reliability.

**Figure 3:**
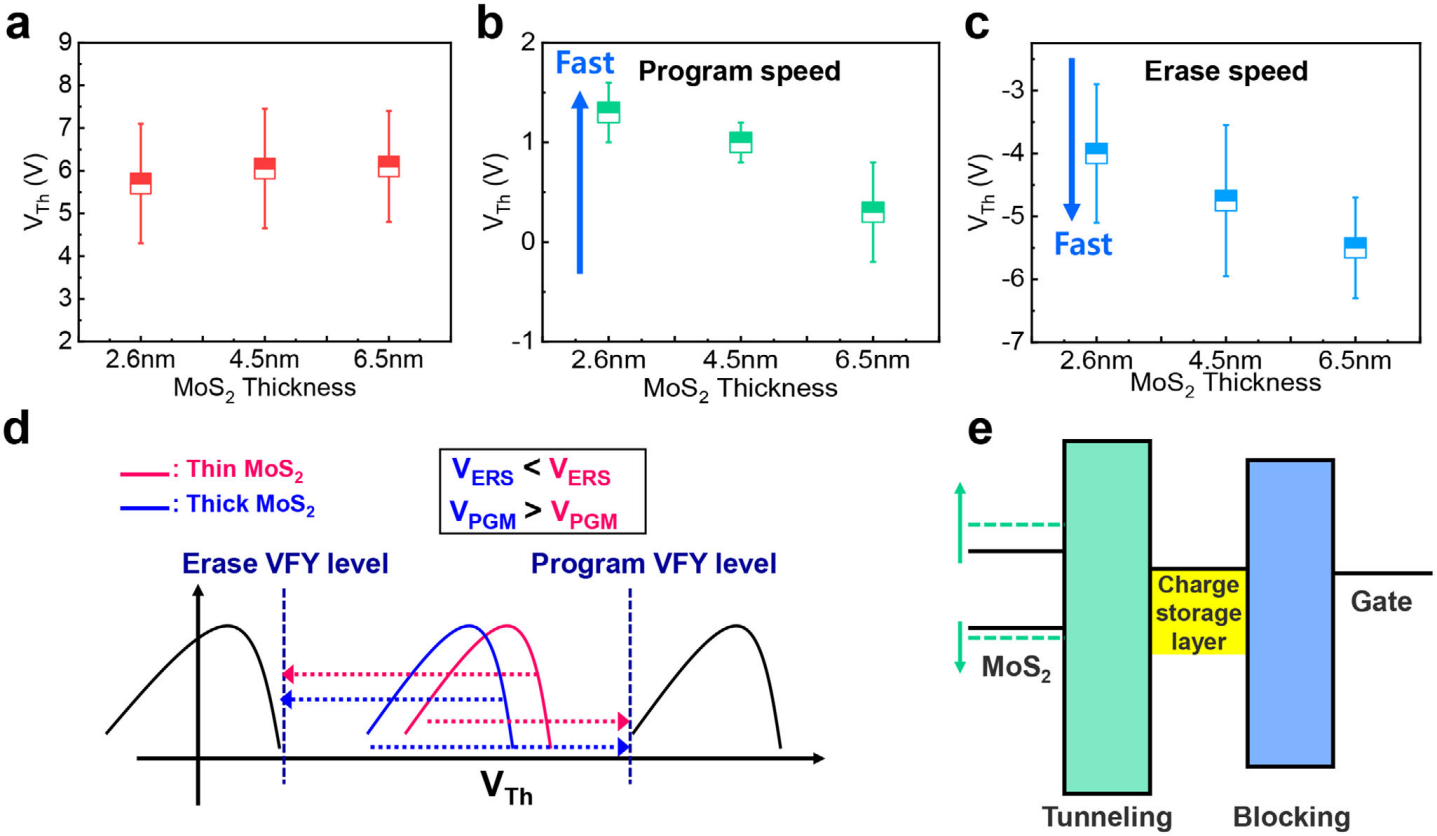
a) Memory window of MoS_2_‐based memory devices as a function of the number of channel layers. b) Program speed and c) erase speed for varying channel thicknesses. As MoS_2_ thickness increases, the program speed is slow while erase speed is fast. d) Model for memory operation speed based on initial *V*_Th_ depending on channel thickness. e) Band diagram of the increased bandgap in the thin channel thickness due to the quantum confinement effect.

**Figure** **3**a shows the memory window of our memory devices as a function of MoS_2_ channel thickness. The memory window was extracted by applying *V*_PGM_ of 14 V and *V*_ERS_ of −14 V with a 10 ms‐pulse width, revealing that it remains independent of the MoS_2_ channel thickness and saturates at ≈6 V. The memory window can be enlarged by increasing the *V*_PGM_ and *V*_ERS_ (Figure S5, Supporting Information). This indicates that the memory window is strongly influenced by the carrier injection rate via voltage‐dependent F‐N tunneling conduction. Note that the relatively large memory window at low operating voltages in MoS_2_ memory devices is due to the high gate‐coupling ratio achieved by the low‐*k* (2.2) pV3D3 tunneling layer, as detailed in Table S1 (Supporting Information), distinguishing it from previous works. Meanwhile, the program and erase speeds are affected by MoS_2_ channel thickness. In the memory industry, program/erase speeds are typically evaluated by the absolute value of the resulting *V*_Th_​, determined by applying identical *V*_PGM_ and *V*_ERS_. This is because in NAND Flash, speed is judged by how close it is to the verify (VFY) level, which is the target *V*_Th_ for program and erase during the incremental step pulse programming (ISPP) scheme, a core technology for multi‐bit 3D NAND Flash.^[^ ^18^ ^]^ Figure 3b,c shows the program/erase speeds for different MoS_2_ channel thicknesses. To minimize the effect of the measurement on the memory state, the read operation was carried out using a narrow voltage sweep range, as shown in Figure S6 (Supporting Information). Thicker channels exhibit slower program speeds but faster erase speeds. The channel thickness‐dependent speed can be explained by two combination factors: 1) initial *V*_Th_ and 2) channel thickness‐dependent bandgap. As previously discussed, a thicker MoS_2_ device shows the reduced *V*_Th_ compared to thinner MoS_2_ device due to poor gate controllability.

As shown in Figure 3d, the reduced initial *V*_TH_ before program and erase operations narrows the distance the *V*_PGM_ must move to the program VFY level while widening the distance the *V*_ERS_ must shift to the erase VFY level, leading to faster program and slower erase speeds. The second factor is related to the tunable bandgap energy by quantum confinement effect in semiconductor material.^[^ ^19^ ^]^ This phenomenon occurs when the dimensions of the semiconductor material are diminished to a scale akin to their de Broglie wavelength, generally within the nanometer range. The quantum confinement in thinner channels widens the bandgap, significantly reducing conduction‐band offset but only slightly altering the valence‐band offset (Figure 3e),^[^ ^19^ ^]^ enabling faster electron injection for programming compared to thicker channels. A low *V*_PGM_, achieved by fast programming speed, helps mitigate program disturbance in 3D NAND Flash—an unintended shift in *V*_Th_ of memory cells in the unselected bit line (BL) —because the gate‐to‐channel potential at the selected WL and unselected BL is low (see Figure S7, Supporting Information, for details on this NAND Flash array operation). This program disturbance widens the programmed *V*_Th_ distributions, causing the read error by narrowing the memory read window. Minimizing program disturbance is vital for multi‐bit per cell technology to realize high‐density 3D NAND Flash. Although the thin MoS_2_ channel layer allows for alleviating program disturbance, the reduced on‐current can degrade the BPD‐induced reliability issue. Therefore, engineering contact resistance to maintain a sufficient on‐current becomes crucial for ultra‐thin MoS_2_ channels.

It is important to compare the program operation of memory cell devices with MoS_2_ and Poly‐Si channel at the same ultrathin thickness. The memory characteristics of MoS_2_ and Poly‐Si channels with a thickness of 1.3 nm were analyzed through TCAD simulation. Both MoS_2_ and Poly‐Si channels‐based devices were calibrated successfully with experimental data (Figure S8, Supporting Information). Due to quantum confinement effects, 1.3 nm‐thick Poly‐Si exhibits a significantly widened bandgap of 5.65 eV, as calculated using the Brus equation (Figure S8, Supporting Information). Although the conduction band offset between 1.3 nm‐thick Poly‐Si and the tunneling layer remains relatively small, the enlarged bandgap leads to a substantial increase in *V*_Th_ required for channel inversion. This indicates that at a thickness of 1.3 nm, the material feature of Poly‐Si resembles that of a dielectric material rather than a semiconductor. In contrast, 1.3 nm‐thick MoS_2_ retains a moderate bandgap (1.9 eV) and a high electron affinity (4.14 eV), which together facilitate efficient electron tunneling and enable stable memory operation (Figure S8f, Supporting Information). These results indicate that MoS_2_ is a highly promising candidate for ultrathin channel applications in high‐density 3D NAND Flash memory.

**Figure 4:**
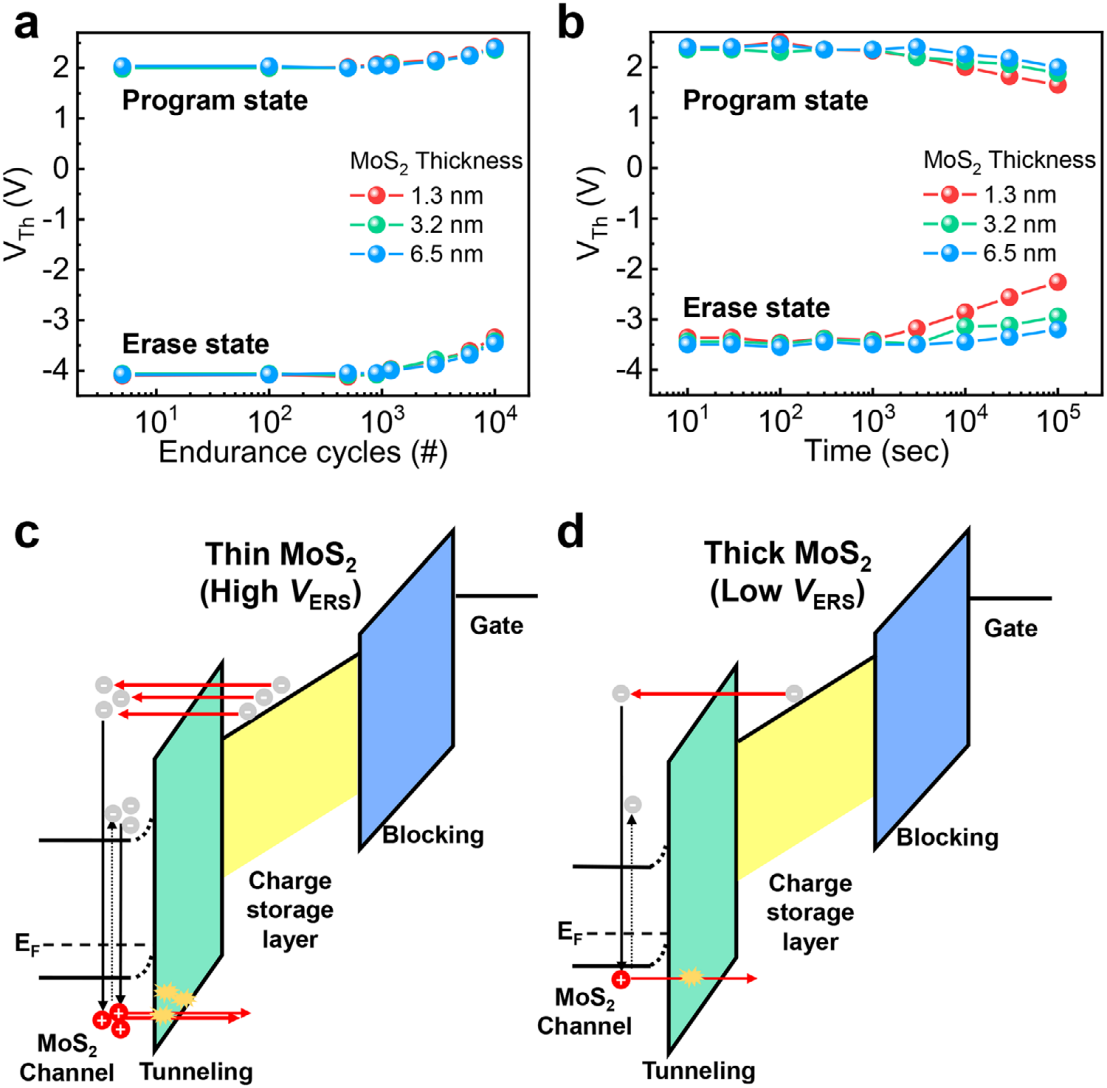
a) Cycling endurance of MoS_2_ memory devices with various channel thicknesses. b) Retention characteristics after cycling endurance tests for MoS_2_ memory devices with different channel thicknesses. Model of AHI degradation for memory devices with c) thin MoS_2_ and d) thick MoS_2_ layers. In thin MoS_2_ devices, the increased *V*_ERS_​ induces more severe AHI‐induced degradation due to a larger number of detrapped electrons from the charge storage layer compared to thick MoS_2_ devices.

Conversely, a fast erase speed can improve the endurance characteristics. It is well established that the enduracne degradation in charge trap flash cell device is attributed to anode‐hole injection (AHI), which generates both bulk traps in the tunneling layer and interface trap between channel layer and tunneling layers.^[^ ^20^ ^]^ The AHI‐induced degradation arises from the hot‐hole injection generated through the impact ionization process by electron detrapping from the charge storage layer under erase operation. Although hole injection is the primary mechanism in erase operations for charge trap flash cell device, the increased *V*_ERS_ values by slow erase speed amplifies electron detrapping from the charge storage layer, worsening reliability. We evaluated the endurance and retention characteristics to investigate device reliability depending on MoS_2_ channel thickness. In the tests, we performed endurance cycling tests for various MoS_2_ thicknesses by applying different *V*_PGM_ ​ and *V*_ERS_ ​ pulses to achieve a program VFY level near +2 V and an erase VFY level of −4 V (each with 10 ms pulse widths). As shown in **Figure** **4**a, there is no noticeable difference in endurance degradation for different MoS_2_ channel thicknesses. However, the retention tests conducted after the endurance tests show notable differences among the devices. While the MoS_2_ memory devices with thicker channels show the lowest *V*_Th_ degradation within 10^5^ s, 1.3 nm‐thick channel memory device exhibits the worst retention characteristics among various channel thicknesses (Figure 4b). This results from the trap generation in pV3D3 tunneling layer via AHI process by slow erase speed. Even though the valence band offset in thin MoS_2_ channel memory device is decreased by quantum confinement effect for efficient hole‐injection, the initial *V*_Th_ increases due to increased controllability which is dominant factor for slow erase‐induced endurance degrdation. Consequently, thin MoS_2_ channel devices experience more electron detrapping from the charge storage layer (Figure 4c), producing large numbers of hot holes and interface traps via impact ionization. A large number of detrapped electrons lead to the generation of numerous hot‐holes and interface traps through impact ionization. The heavy effective mass of these hot holes generates easily the bulk trap in tunneling layer. Although bulk traps form in the tunneling layer, confirming whether these traps lead to reliability issues solely through endurance testing is challenging. However, retention measurements after endurance clearly confirm their effect, emphasizing the importance of evaluating retention to account for endurance‐induced degradation.

**Figure 5:**
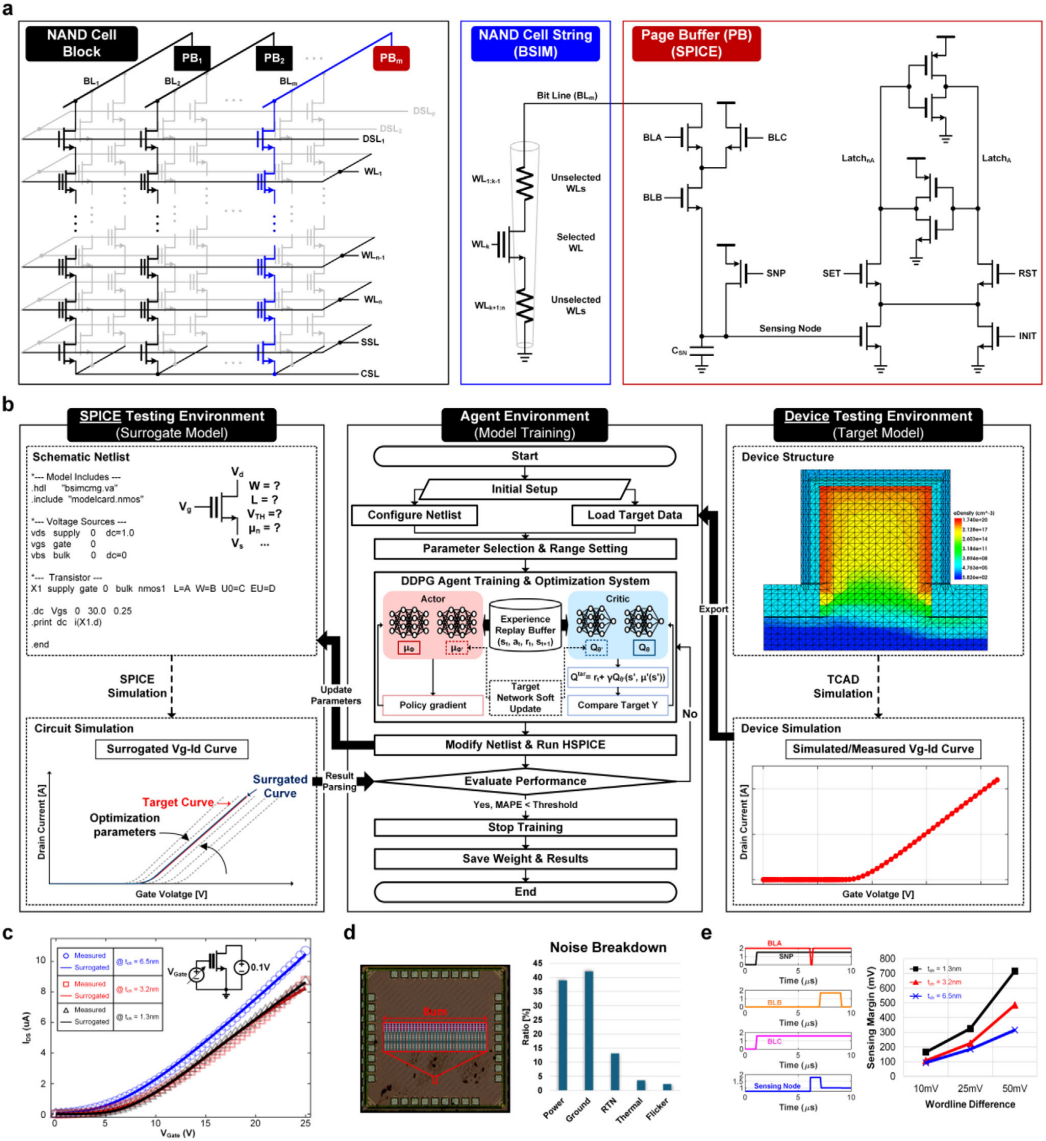
a) Block diagram of the NAND Cell architecture. b) Flowchart of the proposed deep reinforcement learning‐based BSIM Model surrogating framework. c) Comparative analysis of transfer characteristics between target and surrogate models. d) Die micrograph and systematic noise decomposition. e) Sensing margin evaluation under varying word‐line voltage differentials in the comprehensive verification environment.

The verification of cell current sensing capability in relation to MoS_2_ channel thickness in 3D NAND Flash is essential for devising device optimization approach. **Figure** **5**a illustrates the interconnection diagram of a 3D NAND cell block. The NAND cell block structure (*m* × *n* × *p*) comprises multiple strings, where contemporary NAND systems typically implement a page size of 16 KB, corresponding to 16 KB page buffers (*m* = 16 KB). To analyze the sensing characteristics of NAND cell devices within this configuration, it is sufficient to isolate and investigate a single string (BL_m_) and its associated page buffer (PB_m_).^[^ ^21^ ^]^ The NAND cell string can be categorized into unselected WLs (WL_1:k−1_, WL_k+1:n_) and the selected WL (WL_k_), where the unselected WLs can be modeled as resistive elements. The page buffer applies BL voltage to the upper node of the string (BL_m_ node), generating BL current. This current is subsequently converted to voltage through an internal sensing node, and the binary state (On/Off) is determined via a latch mechanism. This sequential process, designated as read operation or verify operation, requires comprehensive consideration of both NAND cell device characteristics and page buffer operational mechanisms for accurate assessment of sensing characteristics. The integration of these parameters enables precise evaluation of the cell device's sensing performance under various operational conditions.

A significant challenge in this verification process lies in the complexity of the testing environment, which necessitates the simultaneous implementation of both NAND cell device characteristics and page buffer circuitry for cell current sensing. This complexity arises from the inherent disparity in verification methodologies: NAND cell device characteristics are typically verified through TCAD device simulation or physical measurements, while circuit operations are predominantly validated through Simulation Program with Integrated Circuit Emphasis (SPICE) simulation frameworks. Previous research has either omitted this integration challenge or attempted to address it through manual implementation of BSIM models.^[^ ^22^ ^]^ To overcome these limitations, this study proposes an advanced verification methodology that employs deep reinforcement learning algorithms to automate and accelerate the process of BSIM model parameter optimization (Figure 5b). This novel approach enables accurate emulation of NAND cell device characteristics—obtained from either TCAD simulations or empirical measurements—within the SPICE simulation environment. The proposed methodology significantly improves the precision and efficiency of testbench implementation by bridging the gap between device‐level and circuit‐level simulations through automated parameter optimization. This integration provides a more comprehensive and accurate verification framework for NAND cell device characterization and circuit operation analysis.

The proposed methodology implements a comprehensive workflow that leverages Deep Deterministic Policy Gradient (DDPG) reinforcement learning to optimize BSIM model parameters through iterative interaction with target model data. The DDPG algorithm formulates parameter tuning as a reinforcement learning problem, where the agent adjusts the model parameters, the environment evaluates the corresponding circuit simulation results, and the reward is determined based on the deviation between simulated and measured device characteristics. By leveraging the DDPG algorithm, which is well‐suited for continuous action spaces, the model efficiently explores the high‐dimensional and nonlinear parameter space. Experimental results demonstrate that the proposed approach achieves superior accuracy compared to conventional numerical optimization methods, offering a more effective solution for BSIM parameter fitting. The entire environment consists of three main components: Device testing environment (Target model), Agent environment (Model training), and SPICE Environment (Surrogate model). The proposed methodology begins by loading reference data (target model data) from TCAD simulations or measurements, which serve as the target for fitting optimization. Subsequently, the agent environment modifies BSIM model parameters in the SPICE netlist to facilitate simulation execution, incorporating the necessary parameter adjustments for each iteration. The DDPG agent is initialized within a carefully configured learning environment, where its actor‐network predicts crucial parameters such as threshold voltage, gate width, gate length, and mobility. The learning process involves running SPICE simulations with the predicted parameters and comparing the resulting curves against the target model curves. This comparison yields a Mean Squared Error (MSE) metric, which provides essential feedback for the reinforcement learning process. The actor‐critic architecture of DDPG enables continuous parameter space exploration while maintaining stable learning progression. The training process continues iteratively until the MSE falls below a predetermined threshold, indicating successful convergence of the SPICE parameters to match the target model data. This automated optimization framework significantly streamlines the traditionally manual process of SPICE parameter calibration while maintaining high accuracy through data‐driven reinforcement learning. Figure 5c shows the curve fitting results using the proposed methodology. Using the experimentally obtained transfer characteristic curve device with MoS_2_ channel thickness of 1.3, 3.2, and 6.5 nm as the target model curve, we performed reinforcement learning‐based curve fitting procedures by setting the mean squared error (MSE) to less than 0.1% in order to represent this with a SPICE model.

Following completion of NAND cell modeling through reinforcement learning, the integrated verification with the BSIM model and page buffer circuit was performed. To distinguish errors originating from the NAND device from those caused by the page buffer, individual page buffer characteristics were comprehensively verified through both simulation and chip fabrication. As shown in Figure 5d, the prototype page buffer chip was fabricated using TSMC 180 nm technology and incorporates a 32‐array page buffer configuration. Power supply and ground noise levels were directly measured on the physical chip by analyzing wrong decision ratios, while other noise components including random telegraph noise (RTN), thermal noise, and flicker noise were characterized through simulation. Through observation of decision errors in both simulation and measurement environments, the root‐mean‐square (RMS) noise voltage corresponding to a wrong decision probability of 16% was calculated. Finally, the sensing margin characteristics of the NAND device during sensing operations were verified. As shown in Figure 5e, the generated voltage at the sensing node was examined as a function of WL level (*V*_WL_), and the program threshold voltage (*V*_Th,PGM_) was designated as the point where the cell current in the given model reaches 30 nA. The page buffer must be capable of making correct decisions even when *V*_Th_ are closely spaced, implying that small differences in *V*_WL_ should result in substantial voltage differentials at the sensing node. Consequently, the sensing margin in NAND page buffer circuits is characterized by the voltage differential generated at the sensing node when considering two distinct WL levels (*V*_Th,PGM_±Δ*V*_WL_), relative to a baseline program threshold voltage (*V*_Th,PGM_). Note that a larger voltage differential between these sensing nodes is advantageous, as it reduces the probability of erroneous decisions due to noise‐induced perturbations. In this work, the sensing margin was evaluated for WL deviations of *V*_Th,PGM_ ± 5 mV (ΔV_WL_ = 10 mV), *V*_Th,PGM_ ± 12.5 mV (ΔV_WL_ = 25 mV), and *V*_Th,PGM_ ± 25 mV (ΔV_WL_ = 50 mV) relative to *V*_Th,PGM_. Notably, the device with the thinnest MoS_2_ layer (1.3 nm) shows the best sensing margin among those with other channel thicknesses. Generally, it apprears that sensing capability will improve by the increased on‐current due to thick MoS_2_ channel. However, as the channel thickness decreases, the sensing margin increases because the transconductance increases even though the on‐current decreases (Figure S9, Supporting Information, for transconductance of MoS_2_ device with various channel thickness). This results from the fully depletion mode operation with enhanced gate controllability in ultrathin body channel MoS_2_ device. Therefore, the results demonstrated that higher NAND cell transconductance—achieved through improved gate control via a thinner channel—leads to enhanced sensing margins. This optimization of the device effectively broadens the operational window for reliable data sensing in NAND Flash memory systems.

In summary, we have thoroughly investigated a high‐mobility MoS_2_ channel material for memory application to address the challenges of high‐density, low‐power on‐device AI. By employing a low‐*k* (≈2.2) pV3D3 tunneling dielectric and discrete Au nanoparticles as the charge‐storage medium, we achieved reduced *V*_PGM_ and *V*_ERS_, thereby improving both endurance and retention characteristics. Through a comprehensive analysis of MoS_2_ channel thickness effects on electrical and memory performance, we established its impact on memory performance—particularly program/erase speeds—and on reliability metrics such as endurance and retention. We further implemented a deep reinforcement learning–driven BSIM parameter optimization to integrate the calibrated MoS_2_ device model with the page‐buffer circuitry, validating the sensing margins at the circuit level. Notably, previous studies on MoS_2_ memory devices have not validated their sensing capabilities due to the absence of a methodology that bridges device‐level characteristics and circuit‐level operations, as shown in **Table** **1**. These findings confirm that MoS_2_‐based 3D NAND Flash can mitigate SCEs and cell current‐induced issues while supporting multi‐bit per cell and reliable data retention. These results offer a feasible strategy for developing next‐generation, energy‐efficient memory systems capable of fulfilling the stringent demands of AI‐centric edge devices.

**Table 1:** Comparison of memory key factors for various reported MoS_2_‐based nonvolatile memory device.

Gate stack	Endurance	Retention	Operating voltage	Coupling ratio	SensingMargin	Refs.
This work	10^4^ cycles	10^5^ s @1‐bit 3000 s@multi‐bit	±14 V	0.61	700 mV per state	
HfO_x_/HfS_2_/HfO_x_/HfS_2_	500 cycles	5000 s@1‐bit	±20 V	N/A	N/A	[24]
HfO_2_/Pt/HfO_2_	5000 cycles	3600 s@1‐bit	±12.5 V	0.17	N/A	[10c]
HfO_2_/AuNPs/SiO_2_	‐	200 s@1‐bit	±100 V	N/A	N/A	[25]
HfO_2_/MLG/HfO_2_	120 cycles	10^4^ s@1‐bit	±15 V	0.17	N/A	[26]
Al_2_O_3_/HfO_2_/ Al_2_O_3_	120 cycles	2 × 10^4^ s@1‐bit	±26 V	0.19	N/A	[27]
HfO_2_/AuNPs/HfO_2_	N/A	10^4^ s@1‐bit	±15 V	0.25	N/A	[28]
hBN/MLG/SiO_2_	1390 cycles	5300 s@1‐bit	±20 V	N/A	N/A	[29]
hBN/MLG/SiO_2_	110 cycles	1400 s@1‐bit	±15 V	0.05	N/A	[30]
SiO_2_/MLG/hBN/PtS_2_ channel	10^5^ cycles	1000 s@multi‐bit	±15 V	0.016	N/A	[31]

## 3 Experimental Section

### Device Fabrication

MoS_2_ channels were formed through mechanical exfoliation onto a 90‐nm‐thick SiO_2_ layer that had been cleaned with piranha solution, paired with a heavily p‐doped silicon wafer. MoS_2_ flakes were obtained from large single crystals using tweezers and directly transferred onto the cleaned wafers. After extraction with scotch tape, the samples underwent annealing process at 300 °C in an environment under 200 torr of H_2_ for 2 h to remove any remaining residual tape contaminants. The metal lines for source and drain electrodes were patterned using conventional photolithography, followed by thermal evaporation of Ti/Au (10 nm/30 nm) in a high vacuum (≈10⁻⁶ torr) and subsequent lift‐off processing. After the MoS_2_ backgate transistor was fabricated, a 13‐nm pV3D3 dielectric tunneling layer was deposited over the MoS_2_ channel via iCVD. A layer of AuNPs was then deposited as the charge storage layer through thermal evaporation. After deposition of the charge trapping layer, a 20‐nm Al_2_O_3_ dielectric blocking layer was formed on the AuNPs layer using atomic layer deposition (ALD). The top‐gate electrode, featuring an underlap design with Cr/Au (5 nm/30 nm), was fabricated through photolithography, thermal evaporation, and lift‐off techniques.

### Chip Fabrication

The page buffer circuit was simulated within the Cadence Virtuoso Custom IC design environment and Synopsys HSPICE simulator. The prototype page buffer of NAND Flash memory was fabricated using 180 nm TSMC CMOS process technology.

### Electrical Measurements

The fabricated MoS_2_ memory devices were evaluated using a Keithley 4200 semiconductor parameter analyzer operated in vacuum and in the absence of light. Furthermore, a B1500 semiconductor parameter analyzer was used to evaluate the memory performances by applying pulse voltages. Before electrical characterization, the devices were subjected to a post‐vacuum annealing process at 100 °C for 2 h to eliminate any absorbed oxygen or moisture from the MoS_2_ surface.

### Characterization

High‐resolution TEM images were obtained using a JEOL ARM300F instrument, with TEM samples prepared for cross‐sectional analysis using a focused ion beam system (FEI Helios Nano Lab 450 HP). Raman and X‐ray photoelectron spectroscopy (XPS) was employed to investigate the effects of the iCVD process on the MoS_2_ channel layer.

## Conflict of Interest

The authors declare no conflict of interest.

## Supporting information



Supporting Information

## Data Availability

The data that support the findings of this study are available from the corresponding author upon reasonable request.
