# Supplementary material for: MoS2 Channel‐Enhanced High‐Density Charge Trap Flash Memory and Machine Learning‐Assisted Sensing Methodologies for Memory‐Centric Computing Systems
Source: Adv Sci (Weinh). 2025 Jun 10;12(32):e01926. doi: 10.1002/advs.202501926 (PMC12407348; doi:10.1002/advs.202501926)
Supplement: Supplementary file 1 — Supporting Information [file ADVS-12-e01926-s001.docx]

Supporting Information

MoS_2_ Channel-Enhanced High-Density Charge Trap Flash Memory and Machine Learning-Assisted Sensing Methodologies for Memory-Centric Computing Systems

Ki Han Kim, Ju Han Park, Khang June Lee, Ji-Won Seo, Yeong Kwon Kim, Junhwan Choi, Min-Jae Seo*, and Byung Chul Jang*

**1. HRTEM image of the gate stack**

**Figure S1.** The enlarged HRTEM image of the gate stack. The scale bar corresponds to 5 nm.

**2. X-ray spectroscopy analysis for iCVD process on MoS_2_ film**


**Figure S2.** X-ray spectrum of MoS_2_ flake before and after pV3D3 deposition via iCVD.

**3. Energy band diagram of MoS_2_-based memory device with low-k tunneling layer**

**
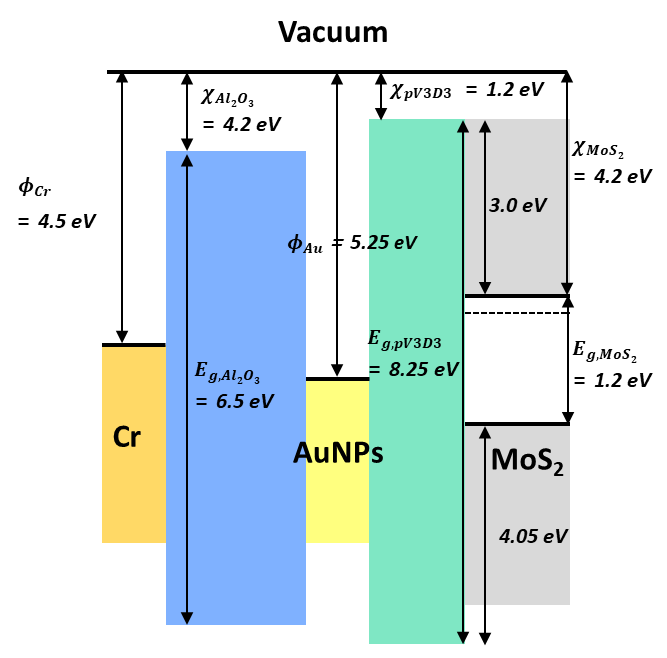
**

**Figure S3.** Energy band diagram of the fabricated MoS_2_ memory device.

**4. Temperature dependent VRH parameter**

**Figure S4.** Gate voltage-dependent variation of VRH parameter *T*_0_. As gate voltage increases, the *T*_0_ decreases.

**5. Tunable memory window by amplitude of pulse voltage**

**Figure S5.** Memory window as a function of gate pulse voltage.

**6. NAND Flash array operation**


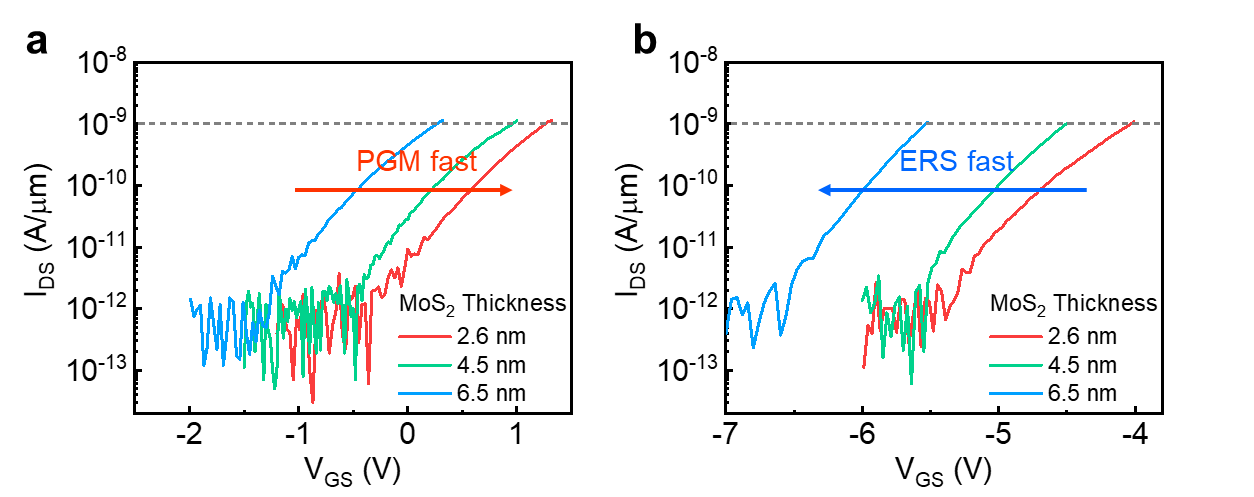


**Figure S6.** (a) Program speed and (b) erase speed as a function of MoS_2_ channel thickness.

**7. NAND Flash array operation**

2D and 3D NAND Flash memory have utilized the Incremental Step Pulse Program (ISPP) scheme to achieve narrow *V*_Th_ distribution by mitigating process and temperature variations.^[1]^ Rather than applying a high *V*_PGM_ pulse to program a cell, ISPP scheme is operated with a series of smaller incremental voltage pulses (Figure S5a). This enables fine control over the charge injected into the charge storage layer, mitigating the risk of over-programming. Each programming step involves applying a voltage pulse and then verifies the cell’s *V*_Th_; if the cell’s *V*_Th_​ is not reached to a target verify level, the next incremental pulse is applied. Once the desired target *V*_Th_​​ is attained, subsequent programming steps are prohibited via a program-inhibit operation. The program-inhibit operation is implemented by boosting the potential of the floated body, achieved by turning off both the drain-side select line (DSL) and source-side select line (SSL) transistors as well as applying appropriate voltages (*V*_PGM_ and *V*_Pass_) to the word lines (WL). This operation reduces the gate-to-body potential (*V*_PGM_ – $\alpha$) where $\alpha$ is the boosted body potential.

During ISPP operation, unintended phenomena can occur, leading to soft program operation. This is called disturbance, which consists of program disturb and pass disturb (Figure S5b). The program disturbance occurs in the cell at the selected WL and unselected BL in a NAND Flash memory array during the programming of a target cell. In contrast, the pass disturbance arises in the cell device at the unselected WL and selected BL. Specifically, the selected WL is biased at *V*_PGM_​, the unselected WL at *V*_Pass_​, the selected BL at 0 V, and the unselected BL at *V*_CC_. The program disturbance results from the lack of the boosted, while the pass disturbance occurs when the large *V*_Pass_ is applied. Thus, program disturbance can be mitigated by the fast program operation via core algorithm and device engineering, indicating that low *V*_PGM_ is essential for reliable 3D NAND Flash operation.


**Figure S7.** (a) ISPP operation methodology of NAND Flash memory array. ISPP operation consists of 1) program and 2) program inhibit operations. (b) Potential condition of cell device for program operation, program disturbance, and pass disturbance.

**8. Analysis of program efficiency of MoS_2_ and Poly-Si channels with a 1.3 nm thickness**

The memory characteristics and energy band diagrams of MoS_2_ and Poly-Si channels with a thickness of 1.3 nm were analyzed through simulation. As shown in Figures 8a and 8b, both MoS_2_ and Poly-Si were calibrated based on experimental data, and then subsequent simulations were conducted to analyze the program characteristics using memory cell structures (Figure S8c). The bandgap and electron affinity of 1.3 nm-thick-MoS_2_ were 1.9 eV and 4.14 eV (Figure 8d), respectively. For 1.3nm-thick Poly-Si, the bandgap and electron affinity were calculated based on the Brus equation ($E_{g}=E_{g,bulk}+\frac{h^{2}}{8d^{2}}\left( \frac{1}{m_{e}^{*}}+\frac{1}{m_{h}^{*}} \right)\approx\frac{7.6}{d^{2}}$ for Si, where d is channel thickness) and the parameters are applied in the simulation (Table S1). As shown in Figure S8e, the bandgap of 1.3 nm-thick Poly-Si increases to 5.65 eV due to the quantum confinement effect, and the electron affinity decreases to 0.905 eV. In order to perform program operation, electrons in the conduction band must be trapped in the charge trap layer through F-N tunneling. However, even though 1.3 nm-thick Poly-Si has a very low conduction band offset between the channel and the tunneling oxide, the *V*_Th_ required for forming the inversion layer of Poly-Si increases due to the larger bandgap than before. In other words, the material characteristic becomes closer to the dielectric than to the semiconductor. As a result, electrons inside Poly-Si cannot tunnel well to the charge trap layer, so it cannot operate as a memory device. On the other hand, 1.3 nm-thickMoS_2_ has a bandgap (1.9 eV) and electron affinity (4.14 eV), making it a suitable channel material for memory operation. Thus, electrons in MoS_2_ can tunnel and trap into the charge trap layer, enabling reliable program operation as a memory device (Figure S8f). In addition, channel thickness scaling is essential for 3D NAND Flash for gate/spacer length scaling enabling for further WL stacking-up. MoS_2_ is a material that can operate as a memory even with a channel thickness of 1 nm, representing a promising material for next-generation high-density memory device.

**Figure S8.** The calibrated TCAD simulation results for (a) thickness of 1.3 nm and 6.5 nm-thick MoS_2_-based devices with our experimental data and (b) 6.5 nm-thick Poly-Si-based device is calibrated with experimental data of 3D NAND Flash memory, where the data are extracted from WL5 in [1]. (c) The MoS_2_ and Poly-Si-based device structure for TCAD simulation. (d) The energy band diagram illustrating the increase in bandgap due to the quantum confinement effect as the MoS_2_ thickness decreases. Program efficiency analysis for 1.3 nm MoS₂ and Poly-Si channel-based device with (e) energy band diagram, and (f) *V*_Th_ characteristics according to *V*_PGM_.

**9. Transconductance of MoS_2_-based device with different channel thickness**

**
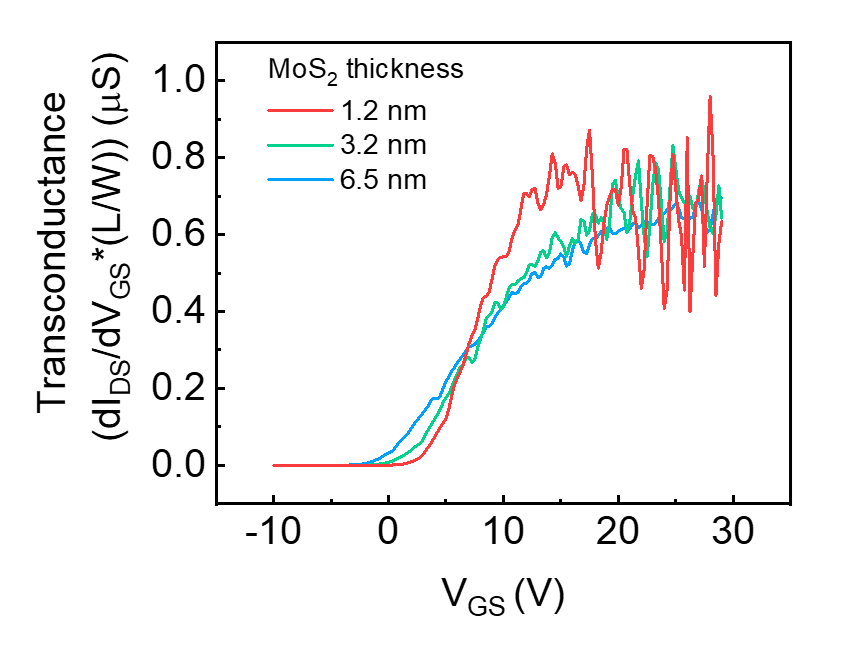
**

**Figure S9.** Transconductance for MoS_2_-based device with different channel thickness as a function of gate voltage.

**10. Material parameter utilized in TCAD calibration**

| **Parameters** | **Value** |
| --- | --- |
| 1.3nm MoS_2_ *ε* [2] | 4.71 |
| 6.5nm MoS_2_ *ε* [3] | 8.31 |
| 1.3nm MoS_2_ Eg_300K [4] | 1.9 eV |
| 6.5nm MoS_2_ Eg_300K [4] | 1.32 eV |
| 1.3nm MoS_2_ χ [4] | 4.14 eV |
| 6.5nm MoS_2_ χ [4] | 4.38 eV |
| MoS_2_ m* [2] | 0.55 m_o_ |
| MoS_2_ *μ* | 30 cm^2^ $V^{-1}s^{-1}$ |
| SiO_2_ Eg_300K | 9 |
| SiO_2_ *ε* | 3.9 |
| SiO_2_ χ | 0.9 eV |
| SiO_2_ m* | 0.42 m_o_ |
| Ti resistivity | 5 x 10^-5^ |
| Ti work function | 4.5 eV |
| 1.3nm Poly-Si Eg [5] | 5.65 eV |
| 1.3nm Poly-Si χ [5-6] | 0.903 eV |

**Table S1.** Material parameters used in TCAD simulation for MoS_2_-device with 1.3 nm- and 6.5 nm-thick channel layers.

**References**

[1] J. Jang, H. S. Kim, W. Cho, H. Cho, K. Jinho, S. I. Shim, Younggoan, J. H. Jeong, B. K. Son, D. W. Kim, Kihyun, J. J. Shim, J. S. Lim, K. H. Kim, S. Y. Yi, J. Y. Lim, D. Chung, H. C. Moon, H. Sungmin, J. W. Lee, Y. H. Son, U. I. Chung, W. S. Lee, presented at *2009 Symposium on VLSI Technology*, 15-17 June 2009, **2009**.

[2] S. B. Desai, S. R. Madhvapathy, A. B. Sachid, J. P. Llinas, Q. Wang, G. H. Ahn, G. Pitner, M. J. Kim, J. Bokor, C. Hu, H. S. P. Wong, A. Javey, *Science* **2016**, 354, 99.

[3] U. Ahuja, A. Dashora, H. Tiwari, D. C. Kothari, K. Venugopalan, *Computational Materials Science* **2014**, 92, 451.

[4] H.-g. Kim, H. J. Choi, *Physical Review B* **2021**, 103, 085404.

[5] S. T. Harry, M. A. Adekanmbi, *International Journal of Research -GRANTHAALAYAH* **2020**, 8, 318.

[6] H. T. Lue, C. C. Hsieh, T. H. Hsu, W. C. Chen, C. P. Chen, C. J. Chiu, K. C. Wang, C. Y. Lu, presented at *2019 Symposium on VLSI Technology*, 9-14 June 2019, **2019**.
